# What can available randomised controlled trials evaluating monitoring strategies tell us about the design and analysis of future trials?

**DOI:** 10.1186/1745-6215-12-S1-A48

**Published:** 2011-12-13

**Authors:** Jacqueline Dinnes, Jenny Hewison, Doug Altman, Jon Deeks

**Affiliations:** 1 Public Health, Epidemiology and Biostatistics, University of Birmingham, Edgbaston, Birmingham, B15 2TT, UK; 2 Leeds Institute for Health Sciences, University of Leeds, Charles Thackrah Building, 101 Clarendon Road, Leeds, LS2 9LJ, UK; 3 Centre for Statistics in Medicine, University of Oxford, Wolfson College Annexe, Linton Road, Oxford , OX2 6UD, UK

## Background

Randomised controlled trials (RCTs) of monitoring regimes present unique challenges. Trials of monitoring evaluate a strategy, “a planned and organised system of repeated assessments and subsequent decisions about additional interventions, such as starting, stopping or modifying treatment” [1] all of which should be specified in advance, and ideally supported by previous research. The complexity of the intervention and consequent potential for “interactions between tests, repeated tests, test results and the decisions based on these results” [1] also necessitates large sample sizes in order to detect an effect on important patient outcomes.

## Objective

To gain an insight into the extent to which monitoring schedules evaluated in RCTs have been informed by prior research and to highlight potential issues for design and analysis of future trials.

## Methods

A review of available RCTs of monitoring regimes intended to allow earlier intervention in people with, or at high risk of, a condition that is likely to recur or progress was conducted. An electronic search of the CENTRAL database was carried out using the keywords (monitor* or serial* or surveill*) in the title or the same keywords in the abstract combined with (biomarker* or marker* or test* or examination* or measure*). RCTs comparing at least one formal monitoring strategy to no formal monitoring, to an alternative monitoring strategy, or to immediate treatment were included. Trials of monitoring where the main purpose was treatment titration, improvement in adherence to a treatment regimen, or evaluating ways of delivering monitoring were excluded. One author conducted the searches, screened the search results for relevant studies and carried out the data extraction. A narrative synthesis will be performed.

## Results

Results will be presented for over 20 RCTs. In addition to information on trial quality, data will be presented on the extent to which the components of the monitoring schedule (testing frequency, threshold for intervention and actions consequent to crossing that threshold) have been specified. The MRC framework for evaluating complex interventions will be used to help inform the extraction of this information [2]. A further focus will be on the types of outcomes assessed by the trials and reporting of sample size calculations.

## Conclusions

The review’s results will be used to inform guidance regarding the future evaluation of monitoring regimes.

## References

[B1] Bossuyt PM Glasziou PP, Irwig L, Aronson JK Evaluating the effectiveness and costs of monitoring Evidence-based medical monitoring. From principles to practice 2008 Oxford: Blackwell Publishing 158 165

[B2] Craig P Dieppe P Macintyre S Michie S Nazareth I Petticrew M Developing and evaluating complex interventions: new guidance 2008 London: Medical Research Council 10.1136/bmj.a1655 PMC2769032 18824488

